# Effects of SARS-CoV-2 Inflammation on Selected Organ Systems of the Human Body

**DOI:** 10.3390/ijms23084178

**Published:** 2022-04-10

**Authors:** Marta Kopańska, Edyta Barnaś, Joanna Błajda, Barbara Kuduk, Anna Łagowska, Agnieszka Banaś-Ząbczyk

**Affiliations:** 1 Department of Pathophysiology, Institute of Medical Sciences, Medical College, University of Rzeszow, 35-959 Rzeszow, Poland; 2 Institute of Health Sciences, Medical College, University of Rzeszow, 35-959 Rzeszow, Poland; ebarnas@ur.edu.pl (E.B.); joanna.blajda@gmail.com (J.B.); 3 Students Science Club “Reh-Tech”, Institute of Medical Sciences, Medical College, University of Rzeszow, 35-959 Rzeszow, Poland; barbarakuduk@wp.pl (B.K.); alagowska7@gmail.com (A.Ł.); 4 Department of Biology, Institute of Medical Sciences, Medical College, University of Rzeszow, 35-959 Rzeszow, Poland; agnieszkabanas@o2.pl

**Keywords:** COVID-19, long-term effects, human body, multi-organ damage

## Abstract

Introduction and purpose of the study: SARS-CoV-2 virus does not only affect the respiratory system. It may cause damage to many organ systems with long-term effects. The latest scientific reports inform that this virus leaves a long-term trace in the nervous, circulatory, respiratory, urinary and reproductive systems. It manifests itself in disturbances in the functioning of the organs of these systems, causing serious health problems. The aim of the study was to review the latest research into the long-term effects of COVID-19 and determine how common these symptoms are and who is most at risk. Based on a literature review using the electronic scientific databases of PubMed and Web of Science on the long-term effects of SARS-CoV-2 infection, 88 studies were included in the analysis. The information contained in the analyzed literature shows that the SARS-CoV-2 virus can cause multi-organ damage, causing a number of long-term negative health complications. Conclusions: There is evidence that the virus can cause long-term complications lasting more than six months. They mainly concern disturbances in the functioning of the nervous, circulatory and respiratory systems. However, these studies are small or short-lasting, and many are speculative.

## 1. Introduction

### 1.1. The Global Pandemic of Severe Acute Respiratory Syndrome Coronavirus 2 (SARS-CoV-2)

Coronaviruses have been known to mankind for decades. At first, they were thought to cause mild infections of the upper respiratory tract. The SARS outbreak occurred for the first time in 2003 and was mastered within six months [1]. There was a large time gap before the second, closely related coronavirus—the Middle East respiratory syndrome coronavirus, which emerged in 2012 [2]. Apart from severe respiratory disease, it also often caused renal failure [3]. They led to the death of a significant number of patients, which showed a significant threat to the situation in the world from these pathogens. As of 30 November 2019, the World Health Organization (WHO) has registered 2494 cases of MERS-CoV worldwide [4]. The 21st century was marked by a pandemic caused by the severe acute respiratory syndrome coronavirus 2 (SARS-CoV-2) virus, the seventh coronavirus known to infect humans, causing the infectious respiratory disease COVID-19 [5]. The first cases of infection were identified and described in December 2019 in the city of Wuhan, Hubei province in central China. Already in mid-January, the virus had spread throughout China, in the second half of February, outbreaks of infections with hundreds of patients broke out in South Korea, Italy and Iran. The virus spread very quickly across all continents, causing a global pandemic officially announced by the World Health Organization (WHO) on 11 March 2020, describing this disease as a serious threat to public health of international scope [6]. The consequences of the COVID-19 pandemic include a significant number of cases and deaths, failure of health systems, mental disorders and unprecedented methods of fighting a pandemic requiring the closure of large sectors of the economy and a drastic reduction of people-to-people contact [7]. A comparison of the SARS-CoV-2 genome sequence and other possible B-coronavirus genomes shows the greatest similarity (96%) of SARS-CoV-2 with the BatCov RaTG13 bat coronavirus strain. This suggests that the SARS-CoV-2 virus naturally evolved from the RaTG13 virus strain transmitted by bats. However, confirmation of its origin requires further research and investigation [8,9].

According to the latest scientific research, the SARS-CoV-2 virus can cause pathology in various organs of the human body. Previous reports refer to selected systems, there are numerical and methodological limitations in research, the effects of infection presented in the literature are often unclear and presumed. For this reason, the authors of this article decided to synthesize the existing research on the effects of infection with this virus and its impact on various organs and organ systems in order to be able to determine which of them affects the most and combine individual reports into a coherent whole [10,11,12,13,14,15,16,17,18,19].

### 1.2. Symptoms of Infection

This disease primarily affects the respiratory system with symptoms such as dyspnea, dry cough and, in severe cases, causes acute respiratory failure. Other common symptoms include elevated body temperature, general weakness, skeletal pain, as well as neurological symptoms such as loss or impairment of the sense of smell and taste, and headaches [4,6,20].

### 1.3. Characteristics of Infections Caused by SARS-CoV-2 Virus

Respiratory failure and acute respiratory distress syndrome (ARDS) are the most common complications of a severe COVID-19 infection. Most hospitalized COVID-19 patients suffer from severe lung injuries, fatal multi-organ failure, and hemolytic anemia [6,21]. The high rates of airborne and contact dispersal, and the persistence of the SARS-CoV-2 virus on the surface explain the rapid spread and difficulty of controlling the development of COVID-19 infection [1,4,6]. According to the currently available scientific evidence, it turns out that despite the resolution of typical symptoms, infection with SARS-CoV-2 virus can leave a long-lasting mark in the form of the damage and dysfunction of many organs [22,23,24]. The SARS-CoV-2 virus, in addition to changes in the lungs, can disturb the functions of almost all organs, but mainly the heart, blood vessels, brain and neurons, as well as the kidneys, liver and reproductive organs [16,22,23,24,25,26,27,28,29]. These effects have been noticed relatively recently and may signal an increased risk of long-term health problems. Complications of viral infection, as well as the possibility of reinfection, due to the lack of immunity and the weakening of the functions of the organs after infection, are becoming a major health problem. While most SARS-CoV-2 infected patients recover very quickly, the potential long-term problems caused by the infection make it imperative to look for and investigate late complications caused by the SARS-CoV-2 virus [22].

### 1.4. Pathogenesis

The virus causes multi-organ damage. It is assumed that this may be due to a systemic increase in inflammatory mediators, i.e., cytokine storm caused by virus infection. The release of a large number of pro-inflammatory cytokines causes a significant increase in the permeability of blood vessels and disorders of blood coagulation, which easily cause organ damage. It is also assumed that the same mechanism causes increased permeability of brain microvessels, so that the SARS-CoV-2 virus can easily cross the blood–brain barrier and enter the nervous system, thus causing a number of disorders, not only typically neurological, but also to other organ systems [23]. An uncontrolled and poorly recognized host response to a cytokine storm is one of the leading causes of severe COVID-19 conditions. In this pandemic scenario, there is an irresistible need to investigate the mechanisms involved in the hyper-inflammatory process and the production of extracellular neutrophil traps (NET) in response to COVID-19, which can lead to a cascade of inflammatory reactions that destroy surrounding tissues including the lungs, heart and kidneys [30]. It is also worth mentioning that SARS-CoV-2 has approximately 80% of the amino acid sequences consistent with SARS-CoV-1 of severe acute respiratory syndrome, which caused over 8000 infections in 2003 [20]. Both viruses use serine transmembrane protease 2 (TMPRSS2) and angiotensin converting enzyme receptor 2 (ACE2) to infect host cells. In addition to the lungs, ACE2 is expressed in many tissues including the cardiovascular system, gastrointestinal tract and liver. Correspondingly, damage to these organs has been observed in COVID-19 patients [6,31]. Observing the effects of previous epidemics of similar viruses can provide a wealth of clues for analyzing the impact of the SARS-CoV-2 virus and predict the potential long-term effects of COVID-19 as they are just emerging in the current pandemic.

## 2. Objective

The aim of the study was to review the latest research on the long-term effects of COVID-19 and determine how common these symptoms are and who is most at risk of them.

## 3. Materials and Methods

### 3.1. Search Strategy and Selection Criteria

We used Preferred Reporting Items for Systematic Reviews and Meta-Analyses (PRISMA) to conduct this systematic review. An electronic search of scientific articles written in English was made in the electronic databases of PubMed and Web of Science (from 2019 to 2021). The compilation of scientific articles was based on the following search strategy and keywords: long-term effects, long-term complications, long-term manifestations, SARS-CoV-2, COVID-19, health complications after COVID-19, cardiovascular system, heart, nervous system, brain, respiratory system, reproductive system, urinary tract, kidney. Terms and keywords were entered in various configurations with an AND or OR hyphen. In this way, a total of 991 works were found. Published case reports, clinical trials and literature reviews were included. All selected studies met the following inclusion criteria: studies published in the years 2019–2021, written in English, referring to the long-term health effects of SARS-CoV-2 infection, and conducted on people over 18 years of age. The exclusion criteria included articles that did not address the effects of SARS-CoV-2 (e.g., treatment only), which covered hospitalization and symptoms and short-term effects, and studies describing deceased patients as well as the effects of SARS-CoV-2 observed on cadavers. In addition, studies on the effects of the pandemic as lockdown, lockdown and isolation on quality of life and health. Animal studies were also excluded. The inclusion and exclusion criteria are also presented in Table 1.

### 3.2. Results

**Figure 1:**
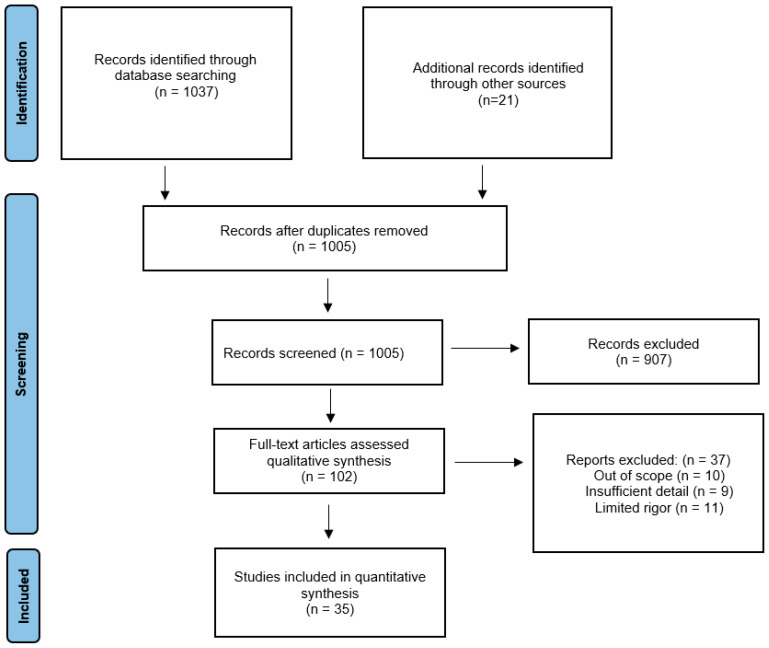
PRISMA Flow diagram.

A total of 1008 articles were identified in the databases, although some were excluded because they analyzed explicit topics that were beyond the scope of this study. After eliminating duplicated studies, 964 studies remained. After selecting by title, abstract and full text, 935 articles were excluded. Ultimately, 88 studies met the inclusion criteria, and they were subjected to the final analysis (Figure 1).

## 4. Circulatory System

According to the Centers for Disease Control and Prevention, elderly patients with coronary artery disease, hypertension or diabetes are more likely to be infected with SARS-CoV-2. Cardiovascular diseases are also associated with a worse prognosis and a more severe course of COVID-19. The virus can also cause heart injuries such as cardiomyopathy and malfunction of the conduction system. Research suggests direct myocardial involvement in some patients [25,32,33].

Overall, infectious myocarditis is the most common cardiac complication of COVID-19 infection. SARS-CoV-2 uses angiotensin converting enzyme 2 (ACE2) receptors to infect host cells, which may cause pneumonia and damage to the heart muscle. High expression of ACE2 receptors in the lungs and heart may increase the risk of cardiac injuries in COVID-19 patients [33]. Tachycardia is also a common cardiovascular complication in COVID-19 patients [34]. Patients with COVID-19 may experience venous and arterial thrombosis due to acute inflammation and hypoxia [26,35]. There are also a large number of late arterial and venous complications.

Cardiovascular complications that are hazardous to health after suffering from SARS-CoV-2 virus infection may develop even several months after the disease and also concern young people, not burdened with additional chronic diseases, which could worsen the course of the infection. The convalescent’s circulatory system manifests its deteriorating condition through, inter alia, arrhythmia, chest pain, and shortness of breath during minimal physical exertion [22]. Patients who have recovered from COVID-19 may experience persistent myocardial involvement as shown by CMR (cardiac magnetic resonance) examination. The main symptoms of CMR included edema, fibrosis, and impaired right ventricular systolic function. The heart condition of COVID-19 patients and survivors must be closely monitored, cardiac CMR can be a sensitive imaging tool coupled with laboratory tests to identify myocardial involvement in COVID-19 patients [10,11,36]. Electrocardiography and echocardiography can also be used to diagnose and predict prognosis in patients with COVID-19 [34] Table 2.

## 5. Nervous System

Numerous studies indicate that the virus is neurotrophic, which means that the virus can enter the nervous system and cause disturbances in its functioning, which also increases the chances of causing long-term effects. The mechanism by which the virus enters the brain is not fully understood. The considerations to date indicate that the S protein of the virus is bound by the ACE2 receptor, which is found in significant amounts in endothelial cells of brain microvasculature. It is believed that this protein may cross the blood–brain barrier. In addition, an infection-induced cytokine storm causes vasodilation in the brain, further facilitating the passage of the virus through the blood–brain barrier into the nervous system. In addition, the virus can enter the brain bypassing the bloodstream. This occurs via the olfactory nerve and travels through the trans-synaptic route to the brain [39]. Neuronal damage caused by COVID-19 may be a driving force behind chronic degenerative diseases of the nervous system. Regardless of its direct or indirect effects, damage to the CNS (central nervous system) following COVID-19 can be permanent [23]. The exact mechanisms by which the virus acts on the nervous system and the underlying causes of the neurological symptoms and effects are unknown. There is only probable speculation that there may be degenerative damage to neurons and glial cells, which are two of the essential elements needed for proper nerve conduction and for the physiological functioning of the brain [40]. In early 2020, the first reports of neuropsychiatric complications during COVID-19 appeared. They related to the occurrence of frequent headaches and dizziness during the infection. Case analysis showed the presence of these symptoms in 36.4% of COVID-19 patients. On the other hand, the group with the severe course of the disease reported disturbed consciousness and cerebrovascular disease [41]. Complications such as encephalitis and encephalopathy have been reported less frequently in the literature [27,28,42,43]. One study estimated that long-term neuropsychiatric consequences may affect approximately 20% of people. The most frequently mentioned were insomnia, depression, anxiety and psychosis. The most frequently presented long-term symptoms include chronic fatigue (53.6), anxiety, depressive symptoms (26.8) and PTSD symptoms (12.2) [28]. First of all, the more known and frequently occurring neurological symptoms caused by SARS-CoV-2 are cognitive disorders, also known as brain fog. These symptoms include general disorientation, confusion, forgetfulness, loss of short-term memory, and significant attention and concentration problems. Headaches and dizziness are also characteristic [44]. This is confirmed by the work on changes in the EEG recording in patients with COVID-19. The information contained in it shows that as a result of infection, new, previously unknown patterns in the EEG recording may appear, which may indicate completely new disorders, or there are waves with specific frequencies characteristic for given neurological diseases and disorders. For example, one of the more common changes in an electroencephalogram are patterns characteristic of encephalopathy, which may explain the appearance of cognitive impairment and a general phenomenon called brain fog [45]. These changes may be caused by a cytokine storm resulting from an excessive response of the immune system, brain hypoxia as a result of which neurons with higher oxygen demand are damaged or by cerebral microcirculation disorders related to infection. Factors such as stress related to infection, dehydration due to difficulties in drinking fluids and fever, sleep disturbances and limited physical activity are also indicated. The individual health situation of the patient is also important [46]. The term brain fog often appears in reports on the chronic effects of COVID-19, and despite the lack of a formal definition, diagnostic categories and inclusion in the international classifications ICD-10 and DSM-V, this concept has been around for a long time. It is used to describe the symptom complexes occurring, inter alia, in Lyme disease, neurosis, Leśniewski-Crohn’s disease, chronic fatigue syndrome, fibromyalgia or celiac disease [47,48,49,50]. A questionnaire study was carried out on a group of 2696 people with documented COVID-19 disease, aged between 18–55 years, a minimum of 3 months after the onset of symptoms. Of these people, 62.3% had long COVID-19 syndrome, and related brain fog occurred in 7.2% of patients, correlating with the female sex and breathing problems in the acute phase of COVID-19 [46]. A very common result of infection are neurological symptoms in the form of long-lasting disturbances of smell and taste. They appear at early stages of the disease and are more common in SARS-CoV-2 infection than in other upper respiratory tract infections. They mainly consist of the loss of these senses, but it may be that they are weakened or appear in the form of parosmia, i.e., feeling a smell completely different from what is actually present, or in the form of a phantosmia, i.e., olfactory hallucinations consisting in perceiving non-existent smells. Post-viral anosmia accounts for up to 40% of cases infected with SARS-CoV-2 [13], with previous coronavirus outbreaks reporting only 10–15% of such cases [51]. There were also reports of 33.9% of those infected with at least one of the olfactory or taste disorders, and 18.3% had both [13]. In another study, 83% of people had anosmia as the first symptom, and 2/3 of them were female. It is worth noting that olfactory disorders occurred without inflammation or symptoms of rhinitis, which leads to the conclusion that the virus directly attacks the mechanisms of odor processing [52]. These symptoms last for about two months [53]. However, there are studies showing that in some infected people, changes in taste and smell persist even 6 months after the infection [52].

It is also considered that SARS-CoV-2 may also contribute to the emergence of mental disorders. This virus activates mechanisms in the body that engage pro-inflammatory cytokine pathways and mechanisms of oxidative stress. They can lead to an imbalance in the regulation of glutamic acid, which contributes to the development of mental disorders [54] Table 3.

## 6. Respiratory System

Due to the fact that the virus primarily affects the respiratory system, it seems justified that it leaves a trace of its infection in the organs of this system. First of all, among convalescents, difficulties in breathing, quick fatigue and significantly reduced endurance to physical exertion are observed. In diagnostic tests, a reduction in the diffusion capacity of carbon dioxide is often observed. In addition, a frequent sight in CT (computed tomography) scans are lung fibrosis or scarring of the respiratory tissues, but depending on the period after hospitalization, the results of diagnostic tests and the observed disorders differ. Nevertheless, these changes may hinder the functioning of the respiratory system and result in the above-mentioned symptoms for a long time after recovery [23,24,59]. Most scientific studies have examined the effects of COVID-19 six months after hospitalization. It has been proven that the vast majority of patients six months after undergoing SARS-CoV-2 show dysfunctions related to the respiratory system. These include mainly accompanying chronic fatigue or severely reduced performance capacity, persistent cough, and exercise dyspnea. In patients examined six months after hospitalization for COVID-19, changes in CT were often associated with the appearance of opacities and fibrosis in the lung tissue. In a large number of patients, a reduced diffusion capacity was also revealed in the functional test [29,60,61,62]. Studies were also carried out one year after hospitalization of COVID-19 patients. Long-term respiratory effects were also observed in them, however, they were associated with a smaller percentage of the subjects and were less intense. The most persistent symptoms are associated fatigue, increased sweating, and tightness in the chest. Patients continued to exhibit reduced performance and reduced diffusion capacity. In the conducted experiments on changes in the CT image, fibrosis was observed in most of the studied patients [63,64,65] Table 4.

## 7. Urinary System

Kidney-related conditions are also included among the long-term effects faced by convalescents. Effects such as proteinuria, oliguria or hematuria are observed [16]. Acute nephritis is frequently observed during infection. It can be the result of multi-organ damage caused by a virus or a direct infection of the virus [17]. An episode of acute nephritis during infection may result in long-term consequences in the form of permanent impairment of kidney function, which in the future may result in kidney failure and the need for dialysis. For this reason, constant monitoring of patients after SARS-CoV-2 infection is necessary [70] Table 5.

## 8. Reproductive System

Current research confirms that SARS-CoV-2 may affect the male reproductive system [71]. SARS-CoV-2 virus attacks host cells through a cell receptor, angiotensin-converting enzyme 2 (ACE2), which provides a binding site for the coronavirus S (Spike) protein. This is important due to the fact that ACE2 plays a dominant role in fertility, especially in oocyte maturation, ovulation and spermatogenesis [72]. This is because the ACE2 receptor is present in the testes and male genital tract, which is where the S protein is expressed while viral infection occurs, suggesting a high probability that it attacks the male reproductive organs during infection [73]. Abnormal levels of sex hormones and deteriorating sperm quality were observed in patients during and after recovery from COVID-19, and severe inflammatory lesions were detected in the testicles. Testicular samples from 2 of the 10 postmortems showed testicular inflammation of unknown origin, but very little data was reported in the study [74]. In addition, in two separate study reports, lesions such as inflammatory damage to the seminiferous tubules with interstitial edema, embolism, inflammatory cell infiltration, and red blood cell effusion were observed in 6 and 11 of 12 patients who died from SARS-CoV-2, respectively [18,75]. In addition, eight patients had varying degrees of impairment in spermatogenesis [75]. In 23 patients hospitalized due to COVID-19, Li et al. also took semen samples. Upon examination, the number of apoptotic cells in the seminal tubules was significantly higher in COVID-19 compared to control cases. It also showed an increased concentration of CD3+ and CD68+ in the interstitial cells of testicular tissue and the presence of IgG in the seminal tubules. The semen of patients hospitalized for COVID-19 showed that 39.1% (*n* = 9) of them had oligozoospermia, and 60.9% (*n* = 14) showed a significant increase in semen leukocytes. Reduced sperm concentration and increased levels of IL-6, TNF-α and MCP-1 in semen were observed compared to the control group [18]. Most importantly, both studies did not detect the SARS-CoV-2 virus in testicular samples with testicular inflammation, suggesting that inflammatory responses rather than viral infections predominated in these lesions [18,75]. The potential impact of fever on sperm quality is indicated. Four patients with moderate infection and fever had reduced semen quality, but whether it was due to inflammation or fever remained unresolved [76]. Infection-related fever has transient adverse effects on spermatogenesis and sperm quality, but usually does not cause irreversible negative effects on male fertility. The adverse effect on sperm is characterized by reduced concentrations, changes in morphology, reduced mobility, and increased DNA fragmentation. These defects may persist for months after the fever has resolved. However, there is no conclusive evidence of the presence of the virus in testicular tissue. The SARS-CoV-2 virus was not present in the testicular tissue of a patient who died in the acute phase of COVID-19 [77]. However, another study showed that the virus can be detected in many organs including the testes [78]. Yang et al., examined testicular samples from 12 deceased COVID-19 patients. The virus was detected in only one patient who had a high viral titer. The SARS-CoV-2 virus was not present in the seminal tubules, but only in the interstitium, and it was not clear whether the virus was derived from the blood or from a testicular infection [18]. In the case of COVID-19, a very important question was whether the virus is sexually transmitted. In six small cohort studies, no viral nucleic acids were found in sperm samples from male patients with mild disease and in the healing phase [18,76,78,79,80,81]. The SARS-CoV-2 virus was not found in the prostate fluid in two independent studies [82,83]. However, one study has shown that SARS-CoV-2 can pass into semen. In this study, out of 23 recovering from COVID-19 and 15 with acute phase patients, 2 (8.7%) and 4 (26.7%) patients, respectively, had positive test for the virus in their semen. This study stands in contrast to previous studies, but it lacks a detailed methodology [84].

All of these studies have a small number of samples. Moreover, the amount of viral RNA in semen was low compared to other tissues and ejaculate collection procedures are prone to contamination. Larger and multicenter studies are needed to draw convincing results regarding the presence or absence of SARS-CoV-2 in semen, especially in patients with early disease or asymptomatic disease. However, according to the available studies, the likelihood of the virus entering the semen in patients in a mild state or in the recovery phase is very low. The injurious effect on male fertility has already been observed during infections with Zika viruses (ZIKV), mumps (MuV) and SARS-CoV-1, while in the case of SARS-CoV-2 the picture of the virus is still incomplete. Long-term surveillance and research will be required to better understand the mechanisms of infection and pathophysiology. However, previous studies of the aforementioned viruses, especially SARS-CoV-1, could provide significant clues regarding the analysis of the effects of COVID-19 on male reproductive performance [85].

Some researchers also link COVID-19 with erectile dysfunction. A study was conducted to assess the odds ratio of the occurrence of erectile dysfunction in patients with a history of COVID-19 with and without comorbidities. Out of a total population of 1,066,108 patients, 9554 were diagnosed with erectile dysfunction. The total number of COVID-19 patients was 7098 (3098 are male) and 146 were diagnosed with both erectile dysfunction and COVID-19. In all cases, the diagnosis of COVID-19 preceded the diagnosis of erectile dysfunction. This means that patients with COVID-19 were 3.68 times more likely to develop erectile dysfunction than patients without COVID-19. SARS-CoV-2 virus infection and erectile dysfunction are strongly related, even after considering known risk factors and demographics [86]. Male gonads may potentially be susceptible to SARS-CoV-2 infection, suggesting caution in observing and evaluating infected men who are planning to conceive. More research is needed to determine if this impairment is temporary or permanent, to elucidate the strategies for SARS-CoV-2 entry into the testes, and how this might affect sperm quality and quantity [87]. The probability of SARS-CoV-2 occurring in the semen of COVID-19 patients is very low, and the semen should rarely be considered a carrier of the SARS-CoV-2 genetic material. However, COVID-19 can cause spermatogenic testicular dysfunction through immune or inflammatory responses. Long-term follow-up is needed in male COVID-19 patients and fetuses conceived during the father’s infection [88]. Information on the involvement of the genitourinary system in recovered COVID-19 patients is very limited. A study was conducted involving men aged 20–50 who were diagnosed with SARS-CoV-2 infection and recovered. Urine samples, prostate secretion, and semen were collected from them in order to detect SARS-CoV-2 RNA. Semen quality and hormonal profiles were analyzed. Seventy-four men were examined. The median interval between the last positive pharyngeal swab RT-PCR test and semen collection was 80 days. The median age was 31 years. The overall sperm quality of COVID-19 patients who recovered was above the WHO lower reference limit. Compared to the healthy control, sperm concentration, total sperm count, and total mobility were all significantly reduced. Moreover, the different clinical types of COVID-19 show no significant differences in semen parameters, but the total sperm count shows a downward trend. Interestingly, people with longer recovery times showed worse data on sperm quality [89] Table 6.

Although research suggests that SARS-CoV-2 may also attack female fertility, no known damage to the reproductive system of a COVID-19 patient has been reported so far, and there is very little information on this topic.

## 9. Digestive System

More and more studies have reported liver damage in patients with COVID-19, and several have shown that patients with COVID-19 have an increased risk of liver dysfunction [90,91,92,93,94]. Most studies showed a higher risk of liver damage in severe COVID-19 than in mildly affected patients, however the exact extent of liver involvement is not clear [95,96]. In a meta-analysis of 12 studies with 1267 patients, the combined incidence of liver injury was 19%, the incidence of ALT elevation was 18%, the incidence of AST elevation was 21% and the incidence of total bilirubin elevation was 6% [97]. Additionally, the level of liver damage appears to correlate with the occurrence of gastrointestinal symptoms. Jini et al. found that the incidence of elevated AST levels was significantly higher in patients with gastrointestinal symptoms than in those without [97]. Xu et al. observed moderate microbial steatosis and mild lobular and portal activity in liver biopsy samples from a patient with COVID-19, which provided evidence of liver damage [98]. It is worth noting that elevated prothrombin time among COVID-19 patients with digestive symptoms is common, and several studies report that thromboembolism is a clinical symptom of COVID-19 [19,99,100,101]. Therefore, liver function and liver enzyme levels should be monitored at an early stage in COVID-19 patients with digestive symptoms. COVID-19 may contribute to the deterioration of liver function in patients who have previously been diagnosed with chronic liver disease and predict an increased risk of severe liver disease. Several studies have shown that the baseline severity of liver disease is strongly associated with the morbidity and mortality associated with COVID-19. Additionally, decompensated cirrhosis, liver cancer, and alcohol-related liver disease are risk factors for adverse COVID-19 outcomes [102,103,104,105,106]. In a multicenter study involving 867 patients with chronic liver disease and COVID-19, 14.0% of patients died, 60.4% were hospitalized, 23% were admitted to the ICU, and 7.7% had decompensated liver function [103]. Another study found that the mortality rate was 32% in COVID-19 patients with pre-existing cirrhosis compared to 8% in those without [107]. Mooni et al. found that 23.3% of patients with cirrhosis and COVID-19 were admitted to the ICU, 17.5% were treated with invasive ventilation, 18.6% received non-invasive ventilation, 4.9% received renal replacement therapy, and 39.8% died [108]. The currently collected data suggest that SARS-CoV-2 infection in patients with cirrhosis appears to be a particularly lethal combination. Compared with patients without baseline liver disease, patients with baseline liver disease have an unfavorable prognosis.

The mechanisms of liver damage in COVID-19 patients are complex. The higher overall mortality among patients with cirrhosis and COVID-19 may be due to the immune dysfunction associated with cirrhosis and metabolic syndrome [109,110], while this requires further studies to be confirmed and investigated Table 7.

## 10. Strengths and Limitations

The studies analyzed in this article provide important information on the risks and pathologies that can be caused by SARS-CoV-2 infection in the body. There are many reliable reports and analyses carried out on large groups of patients subjected to long observations under the supervision of specialists. Many of them clearly indicate which organs are most often abnormal due to infection, and which mechanisms have been disrupted and how [28,29,46,52,55,56,58,60,63,64,65,66,67,68,69]. Nevertheless, a significant proportion of the articles analyzed in this study have some limitations. They mainly concern small groups of respondents, or the research methodology is often unclear, while the conclusions drawn are based on guesswork and are not confirmed. More research is needed because the pandemic is too short-lived to be able to predict with certainty the long-term effects of COVID-19 on the human body. Importantly, there is no distinction made between vaccinated and non-vaccinated patients [11,12,14,16,17,37,54,61,86,89,97].

## 11. Conclusions

There is evidence that the virus can cause long-term complications for more than six months. They mainly concern disturbances in the functioning of the nervous, circulatory and respiratory systems. However, these studies are small or too short-term, and many are speculative. The duration of the epidemic has been too short to be able to conduct reliable studies of the long-term effects of SARS-CoV-2 infection. It is necessary to conduct many years of research on large groups of people that will take into account such factors as: age, gender, demography, level of medical care, the severity of the disease and comorbidities of a person who has had COVID-19.

## Figures and Tables

**Table 1:** Inclusion and exclusion criteria.

Inclusion Criteria	Exclusion Criteria
➢ research into the long-term health effects of COVID-19 ➢ research on the specific effects of COVID-19 on human organs ➢ tests on people over 18 years of age ➢ English language ➢ Research from the years: 2019–2021	➢ research covering hospitalization, symptoms and short-term effects ➢ people under 18 years of age ➢ tests on the deceased ➢ research not on the effects of COVID-19, just symptoms, treatment ➢ the effects of the pandemic as a lockdown, isolation ➢ animal research

**Table 2:** The main effects of COVID-19 on the circulatory system.

Research	Year	Objective	Number of Participants	Material and Method	Results	Conclusions
Shergill S. et al.	2020	Presentation of late complications of COVID-19	1	A case report of a 71-year-old male after COVID-19	A patient fully recovered from COVID-19 with only a persistent altered taste perception reported extreme fatigue, poor appetite with a loss of 5 kg, and sharp left chest pain radiating to the shoulder blade. Upon diagnosis, “blooming aortitis” was found.	According to the authors’ knowledge, this is the first time that aortic inflammation has been associated with COVID-19. This highlights a potential complication in such patients and indicates the need for further research in this area [37].
Putman V. et al.	2020	Assessment of the presence and degree of myocardial damage in patients who have recently recovered from COVID-19 disease.	100	This prospective observational cohort study examined 100 patients recently cured of COVID-19 from the COVID-19 registry of Frankfurt University Hospital between April and June 2020.	In this cohort study of 100 patients who had recently recovered from COVID-19, cardiac magnetic resonance examination revealed myocardial involvement in 78 patients (78%) and ongoing myocarditis in 60 patients (60%), which was independent of the severity of the disease and previous diagnosis.	The results demonstrate the need for continued research into the long-term cardiovascular consequences of COVID-19 [11].
Veyre F. et al.	2020	Presentation of late complications of COVID-19	1	A case report of a 24-year-old man after COVID-19	A 24-year-old man who complained of pain in the right lower limb for a month was diagnosed with frequent femoral artery thrombosis, dilated in the first third of the superficial and deep femoral arteries, associated with thrombosis of the posterior tibial and popliteal arteries. thrombectomy. The patient had no risk factors for thromboembolism.	This case suggests very careful approaches to arterial risk, even if the infection is not severe and the patient is young [12].
Huang L. et al.	2020	Assessing cardiac health in recovered patients from COVID-19 using cardiac magnetic resonance imaging (CMR)	26	26 patients recovered from COVID-19 who reported cardiac symptoms and then underwent CMR examinations were included.	15 (58%) had CMR abnormalities in conventional CMR sequences: myocardial edema was found in 14 (54%) patients and LGE (late gadolinium enhancement) was found in 8 (31%) patients. Reduced right ventricular functional parameters including ejection fraction, cardiac index, and stroke volume/body surface area have been identified in patients with positive conventional CMR results.	Myocardial involvement was found in some patients recovered from COVID-19, which indicates the need to pay attention to the possibility of myocardial involvement in patients recovering from COVID-19 with cardiac symptoms [10].
Brito D. et al.	2021	This study looked at the spectrum of heart abnormalities in student athletes who returned to university campus in July 2020 with COVID-19 without complication.	54	Screening echocardiograms were performed on 54 consecutive student athletes (mean age 19 years; 85% male), who had positive reverse transcription polymerase chain reaction, upper respiratory nasal swab or anti-SARS-CoV-2 immunoglobulin G antibody. Sequential magnetic resonance imaging of the heart was performed in 48 (89%) people.	A total of 16 (30%) athletes were asymptomatic, while 36 (66%) and 2 (4%) athletes reported mild and moderate symptoms associated with COVID-19, respectively. For 48 athletes who completed both imaging studies, 27 (56.3%) subjects were found to have abnormal results.	More than 1 in 3 previously healthy college athletes who recovered from COVID-19 infection showed imaging features of receding pericarditis. Although subtle changes in the structure and function of the heart muscle have been identified, no athlete has shown specific imaging features that could suggest ongoing myocarditis. More research is needed to understand the clinical implications and long-term evolution of these abnormalities in uncomplicated COVID-19 [38].

**Table 3:** The main effects of COVID-19 on the nervous system.

Research	Year	Objective	Number of Participants	Material and Method	Results	Conclusions
Almeria M. et al.	2020	Assessment of the effects of COVID-19 on neurocognitive efficiency	35	The study included 35 patients who recovered from SARS-CoV-2 infection. Appropriate tests and scales, such as TAVEC, WMSIV, and SDMT were used to assess cognitive abilities. The study was carried out from 10 to 35 days after the end of hospitalization	Patients show low scores on the cognitive index. There are also higher scores for anxiety and depression in patients with emerging cognitive disorders	After being infected with the virus, patients may develop cognitive impairment which may persist for more than 30 days after recovery. However, more research is needed [14].
Hopkins C. et al.	2021	Analysis of the long-term influence of SARS-CoV-2 virus infection on olfactory dysfunction	434	A 6-month study was carried out on a group of 613 patients who had symptoms of an olfactory disorder (total or partial loss or parosmia) at the time of entering to the study. 434 subjects completed the study and their olfactory abilities were checked again after 6 months.	44% of respondents reported at least one symptom 6 months after the onset of the infection. 177 subjects after 6 months reported a complete return of the olfactory function, while 12 reported a persistent complete lack of the olfactory ability. The parosmia frequency was 43.1%, which occurred on average about 2.5 months after the loss of smell was reported.	A significant number of people after COVID-19 experience long-term olfactory deficits lasting up to 6 months. Additionally, parosmia is a common complication, even in those patients who report partial restoration of the olfactory function [52].
Otte M.S. et al.	2020	Determination of the olfactory function after infection with SARS-CoV-2 virus using a detailed olfactory test.	91	The study included 91 people with a history of COVID-19 disease. The record of olfactory history was obtained by means of a questionnaire. The olfactory function was assessed by the sniffing sticks olfactory test, and the taste function by the flavor sprays.	80 patients experienced a sudden loss of smell due to COVID-19 infection, while during the study, 33 patients reported impaired olfactory function. At 8 weeks after onset of symptoms, 45.1% of patients were still hyposmic.	In a significant proportion of patients who suffer from loss of smell due to SARS-CoV-2 infection, the symptoms of loss of sense persist for up to 2 months. More research is needed to determine how long the olfactory dysfunction may persist [55].
Vaira L.A. et al.	2020	Risk assessment of long-term persistence of olfactory and taste disorders after SARS-CoV-2 infection	138	The study enrolled 138 patients who underwent COVID-19 and assessed their olfactory and taste functions prospectively over 60 days.	After 60 days of follow-up, 7.2% of patients were still severely dysfunctional. In addition, the risk of developing a long-term disorder becomes significant after 10 days for taste and 20 days for smell.	There is a high risk of long-term persistence of olfactory and taste disorders after contracting SARS-CoV-2 virus. Therefore, if symptoms persist for more than 20 days, appropriate therapy should be instituted [56].
Janiri D. et al.	2020	Determining whether COVID-19 could be a predictor of mental disorder after recovery	61	The study included 61 patients over 60 years of age who recovered after being infected with the SARS-CoV-2 virus. DERS, TEMPS-A-39 and K10 psychological questionnaires were used in the study	29.51% of patients showed emotional disorders. In the group with greater psychological suffering, symptoms of cyclothymic temperament and depression were also shown	There is a high probability of developing emotional disorders as a result of COVID-19 disease in the group of people over 60 years of age [54].
Grover S. et al.	2020	Assessment of mental status, post-traumatic stress disorder (PTSD) and fatigue after recovery from the acute phase of COVID-19.	206	206 adult patients (age > 18 years) who recovered from COVID-19 infection were enrolled in an online survey.	The incidence of anxiety, depressive symptoms and PTSD in the studied sample was 24.8%, 23.8% and 30%, respectively. About three-fifths of the participants (61.2%) had at least one symptom of fatigue. About a quarter of participants (23.7%) reported that they “feel confused and always feel light-headed”, and 38% of patients reported having experienced at least one cognitive problem. The level of perceived self-stigma was observed in 31.1%, 20% reported family-related stigma and 50% reported stigma from neighbors and society.	The study reveals that a significant proportion of patients recovering from COVID-19 experience mental illness, fatigue, cognitive problems and stigma. These issues need to be addressed in routine post-COVID care [28].
Asadi-Pooya A.A. et al.	2021	Study of the frequency of brain fog in patients after undergoing COVID-19 as well as to study potential risk factors.	2696	Adult patients 18–55 years of age with confirmed COVID-19 disease. 2696 patients met the inclusion criteria. A telephone survey was carried out with them, minimum 3 months after recovery.	62.3% of people reported a long COVID-19 syndrome, and 7.2% of patients experienced brain fog associated with it, correlating with the female gender and breathing problems in the acute phase of COVID.	The study reveals that a significant proportion of patients recovering from COVID-19 experience so-called brain fog. These issues need to be addressed in routine post-COVID-19 care [46].
Giacomelli A. et al.	2020	Investigation of the frequency of olfactory and taste disorders in patients with COVID-19.	59	A questionnaire was conducted containing questions about the presence or absence of taste and smell disorders, their type and time of occurrence. 59 patients were interviewed.	Of the 59 patients, 20 (33.9%) reported at least 1 taste or smell disorder and 11 (18.6%) both. Twelve patients (20.3%) presented symptoms before being admitted to the hospital, while 8 (13.5%) experienced symptoms during their hospital stay. Taste changes occurred more frequently (91%) before hospitalization, while after hospitalization, changes in taste and smell appeared with equal frequency. Women reported OTD more often than men.	The study shows that OTDs are quite common in patients with SARS-CoV-2 infection and may precede the onset of a full-blown clinical disease. In the context of the pandemic, more research is needed in non-hospitalized infected patients [13].
McPeake J. et al.	2021	Assessing the long-term psychosocial and physical consequences of severe COVID-19 for patients.	93	A multicenter observational cohort study was conducted; between 3 and 7 months after discharge from hospital. 93 patients who were admitted to intensive care for severe COVID-19 were invited to complete standardized questionnaires on emotional, physical and social recovery, including information about employment.	Emotional dysfunction was common: 46.2% of patients had symptoms of anxiety and 34.4% had symptoms of depression. During follow-up, 53.7% of previously employed patients returned to work; across the socioeconomic gradient, there was a significant difference in return to work, with fewer patients from the poorest areas returning to work (*p* = 0.03). 91 (97.8%) patients with COVID-19 were matched with 91 patients without COVID-19. There were no significant differences in any of the measured results between the two cohorts.	Emotional and social problems are common among people who have survived a severe COVID-19 infection. Coordinated rehabilitation is necessary to ensure optimal recovery of patients [57].
Ahmed G.K. et al.	2021	Study of the long-term effects of post-COVID-19 disease on sleep and mental health, and to discover a possible link between the severity of COVID-19 at the beginning and sleep and mental illness.	182	182 participants were registered six months after being infected with COVID-19 and classified into non-severe (101), severe (60) and critical (20) according to WHO guidelines. All participants were assessed using the “Pittsburgh Sleep Quality Index”, The Post Traumatic Stress Disorder (PTSD) Checklist for the DSM-5 and the Symptom Checklist 90.	Only 8.8% of the respondents had no psychiatric symptoms, while 91.2% had sleep disorders (64.8%), PTSD (28.6%), somatization (41.8%), obsessive-compulsive disorder (OCD) (19.8%), depression (11.5%), anxiety (28%), phobia-anxiety (24.2%), psychoticism (17.6%).	Abnormal sleep, somatization, and anxiety are the most common mental disorders in post-COVID-19. The critical group is often associated with post-traumatic stress disorder, anxiety and psychosis. Being a woman, having diabetes, oxygen support or mechanical ventilation, and high NLR levels all increase susceptibility to mental illness after COVID-19 [58].

**Table 4:** The main effects of COVID-19 on the respiratory system.

Research	Year	Objective	Number of Participants	Material and Method	Results	Conclusions
Sonnweber T. et al.	2021	Assessment of persistent pulmonary lesions after passing COVID-19 60 days and 100 days after virus infection	145	145 patients who underwent COVID-19 were enrolled in the study. 60 days and 100 days from the confirmation of infection, a questionnaire was carried out and clinical examinations of the function and structure of the lungs were carried out	Persistent dyspnea was observed in 36% of the examined patients after 100 days. 21% showed decreased left ventricular function. CT revealed persistent lung pathologies in 63%. Relief of symptoms was observed in comparison to the results after 60 days and after 100 days	Patients undergoing COVID-19 experience long-term changes in the respiratory system, but with time improvement in the function of the respiratory system is observed (picture 3) [66].
van der Sar-van der Brugge S. et al.	2021	Investigation of the effects of falling ill with COVID-19 on lung function, health-related quality of life, and dyspnea.	101	The study involved 101 patients who underwent COVID-19. Six weeks after discharge from the hospital, they underwent a spirometry test, HRQoL questionnaires, test of dyspnea (Borg scale and mMRC), as well as depression and anxiety symptoms (HADS)	21.2% had decreased vital lung capacity. The Borg score was >4 in 19.8% of patients for dyspnea with a score of >2 in mMRC in 23.8%. The median of the HADS scores was 4.0	Most patients undergoing COVID-19, six weeks after discharge from hospital show impaired ability to diffuse. This also translates into a reduced quality of life [67].
Daher A. et al.	2021	The aim of the study was to investigate the impairment of lung function after undergoing COVID-19 and other organ disorders	33	The study included 33 patients hospitalized due to COVID-19. They were examined 6 weeks after discharge. They underwent plethysmography, lung diffusion capacity, gasometry and a 6 min walk test	33% of patients experienced dyspnea and cough, while 45% experienced symptoms of fatigue. Lung function tests showed no significant changes, only a reduced ability to diffuse was observed. Most of the subjects showed a shorter distance in the 6MWT test	No significant functional changes in the respiratory system are observed in patients hospitalized as a result of COVID-19. However, there are often symptoms of fatigue. For confirmation, further studies on a larger group of people are required [15].
Lerum T.V. et al.	2021	The purpose of the study was to describe dyspnea, quality of life, and lung function 3 months after being admitted to hospital for COVID-19	103	The study included a group of 103 patients, including 15 patients in the ICU (intensive care unit) while undergoing COVID-19. The study used the mMRC scale, DLCO spirometry, 6 MWT, pulse oximetry and CT were performed, which were performed 3 months after leaving the hospital	The mMRC score was >0 in 54% and >1 in 19% of participants. Normal spirometry. DLCO was below normal range in 24% of participants. In 25% of the study participants, CT of the chest was opacified and diffusion was reduced. Admission to the ICU was associated with worse results of the tests performed	In most patients, 3 months after the end of hospitalization, the effects after COVID-19 in the form of dyspnea, increased fatigue and changes in the structure of the lungs on CT, the results persist [68].
Wu Q. et al.	2021	Study of pulmonary function changes and CT results in patients with COVID-19 during the recovery period.	54	The study included 54 patients who suffered from COVID-19 and were six months after discharge from the hospital. Symptoms, functional tests and a chest CT scan were performed.	The main symptoms six months after discharge from hospital were fatigue, exercise dyspnea and cough. Almost half of the patients showed lung dysfunction, mainly with restrictive ventilation dysfunction. Eleven patients showed changes in CT scans consisting of turbidity.	Lung dysfunction due to SARS-CoV-2 infection improved over time, but patients did not fully recover six months after discharge from the hospital. Abnormal CT results and lung dysfunction were still observed in some patients (picture 2) [29].
González J. et al.	2021	Investigation of the long-term after-effects in critically ill patients who survived COVID-19.	62	62 patients who were in the ICU as a result of COVID-19 were assessed three months after discharge from hospitalization. Follow-up included symptom and quality of life, anxiety and depression questionnaires, pulmonary function tests, 6MWT (six minute walk test), and chest CT	The most common symptoms that persisted were dyspnea and cough. More than 80% showed a lung diffusion capacity of less than 80%. CT showed abnormal results in 70% of patients in the form of reticular changes or fibrosis.	Three months after hospitalization, lung abnormalities and functional disorders are very common in patients who have undergone COVID-19 [69].
Safont, B. et al.	2021	Assessment of functional respiratory parameters, changes in chest CT and correlation with peripheral blood biomarkers involved in pulmonary fibrosis 2 and 6 months after SARS-CoV-2 infection.	313	313 patients were examined, in whom pulmonary function tests, circulating serum biomarkers, chest radiograph and chest CT were performed.	Patients with altered lung diffusion showed higher levels of biomarkers associated with pulmonary fibrosis. In CT, 66% of patients showed opacities.	Almost half of the COVID-19 patients had impaired pulmonary diffusion six months after discharge from the hospital. Severe patients showed fibrotic changes in CT and elevated serum biomarkers associated with pulmonary fibrosis [60].
Ekbom E. et al.	2021	Evaluation of the long-term effects of COVID-19 on lung function.	60	In 60 patients, 3–6 months after discharge from the hospital, spirometry was performed and the diffusion capacity for carbon monoxide was tested.	Pulmonary dysfunction was found in 52% of the subjects, with the main symptom being reduced diffusion capacity for carbon monoxide.	There is a need to continue research on lung function in patients treated in ICU as a result of SARS-CoV-2 infection [61].
Fang, X. et al.	2021	Evaluation of the long-term after-effects of COVID-19 one year after discharge from hospital among elderly patients.	1233	A multicenter, prospective cohort study was conducted in 1233 eligible elderly COVID-19 patients.	Of the 1233 eligible cases, 630 (51.1%) reported at least one aftereffect. First of all, symptoms that persist one year after discharge from the hospital include fatigue, sweating, and tightness in the chest.	The severity of the disease during hospitalization, age, and follow-up have contributed to the risk of long-term after-effects [63].
Zhou F. et al.	2021	Assessment of the consequences of COVID-19 in patients almost one year after diagnosis, with particular emphasis on the recovery of patients with mild COVID-19.	120	120 patients infected with SARS-CoV-2 were studied. On discharge from hospital, they completed questionnaires assessing symptoms and quality of life. Pulmonary function tests, chest CT scan, 6MWT were also performed.	One year after discharge from the hospital, the measured parameters were checked again. Common symptoms were sleep problems, shortness of breath, fatigue and joint pain. Diffusion disorders were observed in 26% of patients, more than half of the patients showed changes in CT results.	COVID-19 survivors continued to have problems with many systems, including respiratory function, radiography, quality of life, and anxiety and depression [64].
Steinbeis F. et al.	2021	Determination of the reduction in lung function and respiratory quality of life up to 12 months after acute COVID-19.	180	Patients who underwent COVID-19 were studied. They were examined 6 weeks, 3, 6 and 12 months after the onset of symptoms. A chest CT scan was performed, lung function was checked, and symptoms were assessed using a questionnaire.	Pulmonary restrictions and reduced carbon monoxide diffusion capacity were associated with the severity of the disease. The CT results for acute lung involvement was associated with the limitation and reduction of diffusing capacity during the follow-up period.	The severity of respiratory failure during COVID-19 correlates with the degree of lung function impairment and the respiratory quality of life in the year after acute infection [65].

**Table 5:** The main effects of COVID-19 on the urinary system.

Research	Year	Objective	Number of Participants	Material and Method	Results	Conclusions
Peng S. et al.	2020	Description of the two AKI phenotypes and their risk factors and relationship to mortality.	4020	4020 COVID-19 patients hospitalized in Wuhan Third Degree Hospitals, China from 1 January 2020 to 23 March 2020 have been included.	A total of 4020 cases with laboratory-confirmed COVID-19 were included, and 285 (7.09%) of these were identified as AKI. Compared to AKI-early patients, AKI-late patients had significantly higher levels of systemic inflammatory markers. Both AKIs were associated with an increased risk of in-hospital mortality. Only hypertension was independently associated with the risk of early AKI. Whereas age, history of chronic kidney disease, and levels of inflammatory biomarkers were associated with the risk of late AKI.	AKI among COVID-19 patients has two clinical phenotypes that may result from different mechanisms. Due to the increased risk of mortality for both phenotypes, emphasis should be placed on monitoring AKI during COVID-19 [17].
Cheng Y. et al.	2020	Evaluation of the prevalence, risk factors, and prognosis of AKI in adult COVID-19 patients.	1392	Retrospective cohort study of 1392 COVID-19 patients admitted to a university hospital. Clinical characteristics and laboratory data were obtained from electronic hospitalization databases.	A total of 7% (99 out of 1392) of patients developed AKI during hospitalization, including 40% (40 out of 99) within 1 week of admission. In-hospital mortality in patients with AKI stage 1, stage 2 and stage 3 was 62%, 77%, and 80%, respectively.	AKI is rare but is associated with high in-hospital mortality in COVID-19 patients [16].

**Table 6:** The main effects of COVID-19 on the reproductive system.

Research	Year	Objective	Number of Participants	Material and Method	Results	Conclusions
Li H. et al.	2020	Determination of the influence of SARS-CoV-2 infection on male fertility.	23	The study included testicular and epididymal autopsy samples of deceased COVID-19 patients and patients hospitalized for COVID-19. Histopathological examinations were performed on testicular and epididymal samples. Semen parameters and immunological factors were examined in the semen sample.	Autopsy samples from the testes and epididymides showed the presence of interstitial edema, hyperemia, exudate of red blood cells in the testes and epididymides, and thinning of the seminiferous tubules. A reduced concentration of sperm and immunoglobulin factors in semen was observed in comparison with the control group.	Impaired spermatogenesis has been observed in COVID-19 patients, which may be partly explained by an elevated immune response in the testes. In addition, autoimmune orchitis has developed in some COVID-19 patients [18].
Katz J. et al.	2021	Investigation of the relationship between erectile dysfunction and COVID-19 patients.	3098	The odds ratio for erectile dysfunction in COVID-19 patients with and without a history of comorbidities was calculated using the i2b2 patient registry platform.	COVID-19 patients were 3.3 times more likely to develop erectile dysfunction	COVID-19 and erectile dysfunction are strongly related, even after taking into account known risk factors and demographics [86]
Ruan Y. et al.	2021	Assessment of the involvement of the genitourinary system in patients with COVID-19 after recovery.	74	Men aged 20 to 50 years after infection with SARS-CoV-2 were enrolled. Urine, prostate secretion (EPS), and semen were collected for testing for SARS-CoV-2 RNA detection. Semen quality and hormonal profiles were analyzed.	No viral RNA was detected in the body fluids of the genitourinary system. The tested values were within the normal range. Sperm concentration, total sperm count, and total mobility were all significantly reduced.	There was no evidence of direct involvement of the genitourinary system in men after recovery from COVID-19. Patients with a long time (≥90 days) from recovery had a lower total sperm count [89]

**Table 7:** The main effects of COVID-19 on the digestive system.

Research	Year	Objective	Number of Participants	Material and Method	Results	Conclusions
Jin X. et al.	2020	Analysis of confirmed COVID-19 cases with gastrointestinal symptoms in Zhejiang Province to determine epidemiological, clinical and virological characteristics.	74	74 confirmed COVID-19 cases with gastrointestinal symptoms were analyzed using multivariate analysis for their risk of severe/critical disease transition.	COVID-19 patients with gastrointestinal symptoms, in 17 (22.97%) and 23 (31.08%), showed severe and critical conditions, respectively, much more severe than in patients without gastrointestinal symptoms, 47 (8.14%) and 118 (20.45%). Among COVID-19 patients with gastrointestinal symptoms, 29 (39.19%), 23 (31.08%), 8 (10.81%) and 16 (21.62%) had significantly higher rates of fever > 38.5 °C, fatigue, shortness of breath.	Cases of COVID-19 with gastrointestinal symptoms may predispose to a more severe course of the disease and more attention should be paid to such patients [97].
Chen S. et al.	2021	Analysis of the connection between clotting dysfunction and liver injury in patients with COVID-19.	74	A retrospective analysis of 74 COVID-19 patients was performed. According to the coagulation function, 27 cases were included in the coagulopathy group and 47 cases in the control group. A case-control study was conducted to analyze the correlation between the incidence of clotting dysfunction and liver damage in patients with COVID-19.	Alanine aminotransferase (ALT) and aspartate aminotransferase (AST), markers of liver injury, were positively correlated with coagulopathy (*p* = 0.039, OR 2.960, 95% CI 1.055–8.304; *p* = 0.028, OR 3.352, 95% CI 1.137–9.187). The results showed that the presence of clotting dysfunction did not statistically correlate with the severity of COVID-19.	Coagulation disorders in patients with COVID-19 are closely related to liver damage. Longer course of the disease may result in a vicious cycle of coagulopathy and liver damage [19].
Kim D. et al.	2020	Identification of factors associated with adverse outcomes in CLD patients who have acquired the new Coronavirus-2019 (COVID-19).	867	A multicenter, observational cohort study was conducted in 21 institutions in the United States (USA) of 867 adult CLD patients with a laboratory-confirmed diagnosis of COVID-19.	All-cause total mortality was 14.0% (*n* = 121) and 61.7% (*n* = 535) had severe COVID-19. Patients with diarrhea or nausea/vomiting were more likely to have severe COVID-19. Hepatic factors with an independent risk of increased overall mortality were alcohol-related liver disease (ALD), decompensated cirrhosis and hepatocellular carcinoma. Other factors were age, diabetes, high blood pressure, chronic obstructive pulmonary disease, and current smoker, Hispanic ethnicity and decompensated cirrhosis of the liver.	ALD, decompensated cirrhosis and HCC are predictors of higher overall mortality among patients with CLD and COVID-19. The results will enable risk stratification and personalization of management of patients with CLD and COVID-19 [103].
Kuderer N.M. et al.	2020	Describing the outcomes of a cancer patient cohort and COVID-19 and identification of potential prognostic factors for mortality and severe disease.	928	Data were collected from patients 18 years and older with active or history of cancer, confirmed to be infected with the acute respiratory distress syndrome 2 (SARS-CoV-2) coronavirus (SARS-CoV-2) from the USA, Canada and Spain.	In logistic regression analysis, independent factors associated with the increased 30-day mortality, partially corrected, were: increased age, male gender, smoking status, number of comorbidities, Eastern Cooperative Oncology Group performance status 2 or greater, active. Race and ethnicity, obesity status, type of cancer, type of cancer therapy, and recent surgery were not associated with mortality.	In cancer and COVID-19 patients, 30-day all-cause mortality was high and was associated with general risk factors and risk factors specific to cancer patients. Longer follow-up is needed to better understand the impact of COVID-19 on outcomes in cancer patients, including the ability to continue with specific cancer therapies [104].
Bajaj J.S. et al.	2021	Comparison of the results between patients with cirrhosis and COVID-19, and patients with cirrhosis alone and COVID-19 alone.	272	Multicenter study of hospitalized patients with cirrhosis + COVID-19 compared to age/sex matched patients with COVID-19 alone and cirrhosis alone.	37 patients with cirrhosis + COVID-19 were compared with 108 patients with COVID-19 and 127 patients with cirrhosis from seven origins. Race/ethnicity was similar. Patients with cirrhosis + COVID-19 had a higher mortality compared to patients with COVID-19, but not between patients with cirrhosis + COVID-19 and patients with cirrhosis. Patients with cirrhosis + COVID-19 compared to patients with COVID-19 alone had equivalent respiratory symptoms, changes in the chest, and transfer and ventilation rates to the intensive care unit.	Patients with cirrhosis + COVID-19 had similar mortality compared to patients with cirrhosis alone, but higher than patients with COVID-19 alone. The CCI was the only independent mortality predictor for the entire matched cohort [106].
Marjot T. et al.	2021	Determining the impact of COVID-19 on patients with pre-existing liver disease that is currently poorly defined.	745	Data on 745 patients with CLD and SARS-CoV-2 (including 386 with cirrhosis and 359 without) were collected by 2 international registries and compared with data for patients without CLD and SARS-CoV -2 from the UK hospital network.	The mortality rate was 32% in patients with cirrhosis compared to 8% in patients without cirrhosis.	In the largest such cohort to date, we have shown that baseline liver disease severity and alcohol-related liver disease are independent risk factors for death from COVID-19. These data have important implications for risk stratification of CLD patients worldwide during the COVID-19 pandemic [107].

## Data Availability

Not applicable.
